# Electrical Stimulation of Denervated Rat Skeletal Muscle Retards Capillary and Muscle Loss in Early Stages of Disuse Atrophy

**DOI:** 10.1155/2017/5695217

**Published:** 2017-04-13

**Authors:** Kouki Nakagawa, Hiroyuki Tamaki, Keishi Hayao, Kengo Yotani, Futoshi Ogita, Noriaki Yamamoto, Hideaki Onishi

**Affiliations:** ^1^Institute for Human Movement and Medical Sciences, Niigata University of Health and Welfare, Niigata, Japan; ^2^National Institute of Fitness and Sports in Kanoya, Kanoya, Japan; ^3^Niigata Rehabilitation Hospital, Niigata, Japan

## Abstract

The purpose of the present study is to investigate the effects of low-frequency electrical muscle stimulation (ES) on the decrease in muscle mass, fiber size, capillary supply, and matrix metalloproteinase (MMP) immunoreactivity in the early stages of denervation-induced limb disuse. Direct ES was performed on the tibialis anterior muscle following denervation in seven-week-old male rats. The rats were divided into the following groups: control (CON), denervation (DN), and denervation with direct ES (DN + ES). Direct ES was performed at an intensity of 16 mA and a frequency of 10 Hz for 30 min per day, six days a week, for one week. We performed immunohistochemical staining to determine the expression of dystrophin, CD34, and MMP-2 in transverse sections of TA muscles. The weight, myofiber cross-sectional area (FCSA), and capillary-to-fiber (C/F) ratio of the tibialis anterior (TA) muscle were significantly reduced in the DN group compared to the control and DN + ES groups. The MMP-2 positive area was significantly greater in DN and DN + ES groups compared to the control group. These findings suggest beneficial effects of direct ES in reducing muscle atrophy and capillary regression without increasing MMP-2 immunoreactivity in the early stages of DN-induced muscle disuse in rat hind limbs.

## 1. Introduction

Limb disuse due to denervation (DN) causes musculoskeletal atrophy accompanied by a reduction in muscle tissue capillary supply. In particular, steep decreases in muscle mass and force generated by the muscle are observed in early-stage disuse atrophy. Significant decreases in muscle mass and fiber size occur within one week of denervation, and by two weeks the tibialis anterior (TA) muscle atrophies to about 50% of the control [1–3]. Muscle fiber atrophy is accompanied by increased capillary density, reduced capillary-to-fiber (C/F) ratio, and decreased capillary diameter after hind limb unloading and denervation [4–6]. Capillaries supply muscle fibers with oxygen and nutrients and remove waste metabolic products. Capillary density is also reduced because less blood flow is required to meet the metabolic demands in the atrophic muscle. Therefore, therapeutic approaches that enhance blood flow induced by muscle contractions might ameliorate muscle atrophy and capillary loss during the early stages of DN-induced disuse.

Electrical muscle stimulation (ES) has been utilized as a therapeutic intervention and a functional substitute for voluntary muscle contraction in patients with spinal cord injury (SCI) [7] and long-standing denervation. Some clinical studies have found that direct ES to denervated muscles increases muscle mass and average fiber diameter [8, 9]. Moreover, the early application of ES treatment following orthopedic surgery may effectively attenuate loss of muscle strength and improve functional performance [10]. In contrast, other preclinical studies have demonstrated no beneficial effects of chronaxie-based ES (frequency: 20 Hz; pulse duration: twice the chronaxie value; 3 s on, 6 s off) on nerve crush-induced muscle fiber atrophy and muscle excitability [11]. In fact, chronaxie-based ES has been reported to induce muscle fibrosis and atrophy in denervated muscles [12]. Furthermore, although high-intensity (16 mA) ES was effective in retarding denervated muscle atrophy, it may have adverse effects on the regeneration of nerve terminals in the neuromuscular junction and/or the membrane systems involved in excitation-contraction coupling [13]. The effects of ES on muscle atrophy appear to be influenced by the type of disuse model used, as well as the nature of the experimental ES regimen, which can vary in the intensity, frequency, and number of contractions [14–16].

In recent years, it has been reported that matrix metalloproteinases (MMPs) play an important role in the homeostasis and maintenance of myofiber functional integrity; MMPs break down the extracellular matrix (ECM) and regulate skeletal muscle cell migration, differentiation, and regeneration [17]. MMPs are also essential for the angiogenic response to physical activity, which is a complex process thought to be regulated by a balance of proangiogenic and antiangiogenic factors [18]. At the initial stage of capillary sprout formation, the continuous basement membrane, which is composed of type IV collagen and laminin-rich connective tissue membrane underlying endothelial cell layers, must first be dissolved by proteinases before the migration and proliferation of distinct endothelial cell populations occur. MMP-2 (66 kDa, type IV collagenase, gelatinase A) activity is important in the early stages of endothelial cell morphogenesis/capillary formation, as MMP-2 and MMP-9 are responsible for the fragmentation of basement membrane type IV collagen 9 [19, 20]. Furthermore, based on inhibition studies of MMP-2, ablation of MMP-2 may impair not only angiogenesis, but also the growth of regenerated muscle fibers via decreased VEGF and nNOS expression [21]. Therefore, MMP-2 may also play a significant role in myogenesis and muscle regeneration [22, 23].

The activity of MMP-2 has been reported to increase following DN and limb immobilization [24, 25] or muscle stretching in the denervated muscle [26]. Similarly, chronic ES and exercise training elevate MMP-2 expression to various levels depending on intensity [27, 28]. On the other hand, other studies show that ES treatment (20 Hz for 3 s followed by 6 s of rest, repeated for 20 maximal contractions, applied every 48 h for 28 days) does not alter MMP-2 activity [25], indicating that discrepancies might occur among interventions with different conditions. To our knowledge, the histomorphological effects of low-frequency ES on muscle fibers, capillaries, and MMP-2 immunoreactivity in early-stage DN-induced hind limb disuse have yet to be studied, although some studies have demonstrated that even low-frequency ES (10 Hz, incomplete tetanus) reduces DN-induced muscle loss [13, 16]. Thus, it is important to explore the potential use of low-frequency ES (e.g., 10 Hz) to maintain both muscle mass and capillaries. We hypothesize that low-frequency ES reduces muscle atrophy and capillary loss in early stages of DN-induced limb disuse.

The purpose of the present study is to investigate the histomorphometric effects of low-frequency ES on the decrease in muscle mass, fiber size, capillary density, and MMP immunoreactivity in the early stages of DN-induced limb disuse.

## 2. Materials and Methods

Twenty-two male Fischer 344 rats (CLEA, Tokyo, Japan) were individually housed in standard cages at constant temperature (23 ± 2°C), humidity (55%  ± 5%), and 12 h light-dark cycles. Rats were provided with CE-2 rodent chow (CLEA) and water ad libitum. Seven-week-old rats were randomly assigned to one of the following groups: age-matched controls (CON, *n* = 8), denervation with direct ES (DN + ES, *n* = 8), or denervation without ES (DN, *n* = 6). The sample size (*n* = 6-7/group) was calculated with reference to FCSA data from previous studies from our laboratory, using the following formula [6, 29–31]:

(1) $$\begin{array}{c} n=\frac{\left(r+1\right)}{r}\times \frac{\sigma^{2}{\left(Z_{\alpha /2}+Z_{\beta }\right)}^{2}}{\Delta^{2}}, \end{array}$$

where *r* is the ratio of the larger group to the smaller group, *σ* is the standard deviation, Δ is the effect size, *α* = 0.05, *β* = 0.2 (for 80% power with 95% confidence), *Z*_*α*/2_ = 1.96, and *Z*_*β*_ = 0.84.

All animal manipulations and protocols were carried out in accordance with the guidelines presented in the Guiding Principles for the Care and Use of Animals in the Field of Physiological Sciences, published by the Physiological Society of Japan. All procedures were approved by the Animal Committee of the National Institute of Fitness and Sports and the Animal Committee of Niigata University of Health and Welfare.

The rats in the DN and DN + ES groups were anesthetized with an intraperitoneal injection (50 mg/kg) of sodium pentobarbital. An incision was made on the skin covering the left buttock and the sciatic nerve was exposed and separated from the surrounding tissue. The sciatic nerve was frozen for 5 s using a 5 mm diameter stainless steel rod cooled in liquid nitrogen [13, 16, 32–35]. This freezing procedure uniformly damages nerve fibers, similar to nerve crushing, cutting, or transection with a suture [13, 32, 34, 35]. Evoked electromyography was performed with ES at a location proximal to the freezing site of the sciatic nerve to confirm the efficacy of TA muscle denervation [36].

On the day after surgery, the left TA muscles of the DN + ES rats under isoflurane inhalation anesthesia (2%) were percutaneously electrically stimulated. The stimulation protocol was delivered as previously described [16, 37]. Bipolar silver surface electrodes (3 mm diameter) were briefly attached to the shaved anterior surface of the left leg. ES was performed using electrostimulators with an isolator (SEM-4201, Nihon Kohden, Tokyo, Japan) for 30 minutes a day, six days a week, for one week at an intensity of 16 mA, a frequency of 10 Hz, and a pulse width of 250 *μ*s. The ES regimen comprised 2 s of stimulation followed by 6 s of rest. Muscle contractions induced by ES at 10 Hz are characterized by minimal summation during twitch contraction [16]. The ES regimen used in the previous study (10 Hz for 2 s followed by 6 s of rest, repeated for 225 cycles over 30 min) caused as many as 4500 instances (10 × 2 × 225) of muscle contraction, mechanical stimuli, or both. One week after denervation, rats in the control, DN, and DN + ES groups were anesthetized with sodium pentobarbital (50 mg/kg body weight). TA, extensor digitorum (EDL), and soleus (Sol) muscles were extracted and weighed. Left TA muscle samples were mounted on a piece of cork with OCT compound and frozen in isopentane cooled in liquid nitrogen for histological analysis. Samples were stored at −80°C until use.

The TA muscle samples were cut into 10 *μ*m cross sections with a cryostat (CM3050S, Leica, Germany) at −20°C and mounted on silanized slides for immunohistochemical and haematoxylin and eosin staining. After fixing with ice-cold 4% paraformaldehyde for 15 min, sections were blocked at room temperature for 1 h with 10% normal goat serum (NGS) and 1% Triton X-100 in PBS and then washed twice in PBS for 5 min. Next, sections were incubated in 5% NGS and 0.3% Triton X-100 in PBS for 16–20 h at 4°C with a primary antibody against dystrophin as a plasma membrane marker (1 : 500 dilution, Abcam, Tokyo, Japan), CD34 as a marker for endothelial cells (1 : 250 dilution, Abcam, Tokyo, Japan), and MMP-2 (1 : 2000 dilution, Abcam, Tokyo, Japan). The sections were washed several times with PBS, incubated with Alexa Fluor 488 or 568 conjugated secondary antibody (1 : 500 dilution, Abcam, Tokyo, Japan) in 5% NGS and 0.1% Triton X-100 PBS for 1 h at room temperature, and then mounted with Vectashield mounting medium. Images of TA muscle sections were obtained using a fluorescent light microscope (BX60; Olympus, Tokyo, Japan) and a CCD camera (DP72; Olympus, Tokyo, Japan). Digital images at 200x magnification were used to determine the cross-sectional area (CSA) of muscle fibers and the capillary number in each TA muscle. The FCSA of at least 75 fibers in each muscle were measured using Image-Pro Premier software (Media Cybernetics, Rockville, MD, USA). The total number of transverse capillaries and fibers in four random 154 × 205 *μ*m fields was manually counted to determine the capillary density (CD) and the C/F ratio [6, 38]. The MMP-2 positive areas were measured within the area of interest (386 × 386 *μ*m) in each TA muscle.

Data are presented as mean ± SD. Data sets were analyzed using one-way analysis of variance (ANOVA) followed by either the Bonferroni post hoc test or the Kruskal-Wallis test followed by Steel-Dwass multiple comparison tests, depending on the normality of the data distribution [39]. *P* values less than 0.05 were considered significant.

## 3. Results

**Figure 1:**
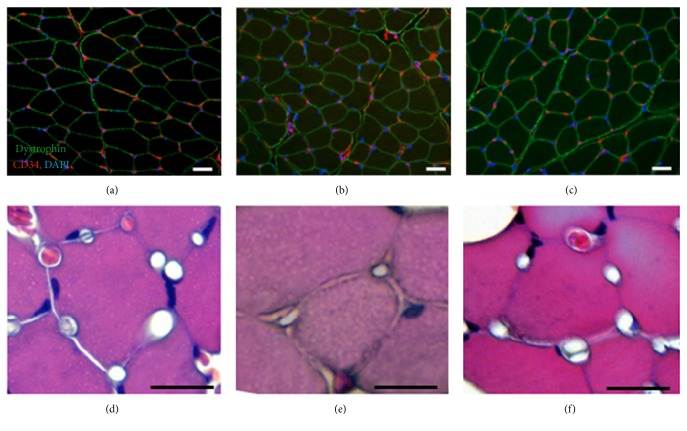
Immunohistochemical staining (a–c) for dystrophin (green), CD34 (red), and haematoxylin and eosin staining (d–f) in the CON (a, e), DN (b, e), and DN + ES (c, f) groups in TA muscles. Scale bar = 25 *μ*m.

Body weight did not significantly differ between the groups (Table 1), though TA muscle weight significantly decreased relative to body weight after denervation (*P* < 0.05). Relative TA muscle weight was significantly higher in the DN + ES group compared to the DN group (*P* < 0.05). Relative EDL muscle weight was significantly lower (*P* < 0.05) in the DN and DN + ES groups than in the control group, but no significant difference was observed between the DN and DN + ES groups (Table 1).  Figure 1 shows representative images of TA muscle FCSA in the control, DN, and DN + ES groups. The CSA of TA muscle fibers were significantly smaller in the DN group than in the control and DN + ES groups (*P* < 0.05, Table 1).

**Figure 2:**
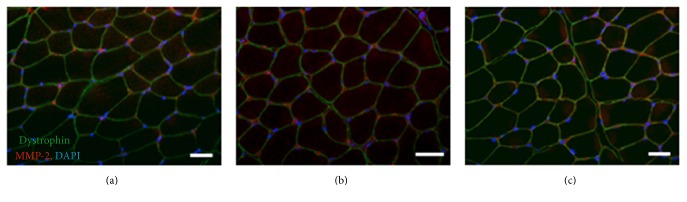
Immunohistochemical staining (a–c) for dystrophin (green), MMP-2 (red), and TA muscles in CON (a), DN (b), and DN + ES (c) groups. Scale bar = 25 *μ*m.

We examined the expression and localization of CD34, a capillary endothelial marker, and dystrophin, a sarcolemmal membrane marker (Figures 1(a)–1(c)). Immunohistochemical staining revealed that CD34-positive capillaries were observed around the dystrophin-positive myofibers. The C/F ratios in the control, DN, and DN + ES groups were 1.22 ± 0.33, 0.73 ± 0.20, and 1.17 ± 0.26, respectively. The C/F ratio was significantly lower (*P* < 0.05) in the DN than in the control and DN + ES groups (Table 1). No significant differences were observed in capillary density between groups. MMP-2 was immunolocalized at the periphery of myofibers (Figure 2), and the MMP-2 positive area was significantly greater (*P* < 0.05) in the DN and the DN + ES groups compared to the control group (Table 1).

## 4. Discussion

The current study presents new data on the effects of low-frequency ES in early stages of disuse atrophy on denervated rat muscles. The results show that ES reduced the decrease in muscle weight, FCSA, and C/F ratio in early-stage DN-induced muscle disuse. The MMP-2 positive area increased in denervated TA muscle, whereas it remained unchanged in denervated TA muscle that was treated daily with ES.

Our results show that EDL and Sol muscle weight were lower in the DN and DN + ES groups than in the control group, and there was no difference between the DN and DN + ES groups. The EDL lies in a deeper layer than the TA, but both muscles are innervated by the tibial nerve, which is a branch of the sciatic nerve. Thus, the present data suggest that successful sciatic nerve denervation had no differential effect on the DN and the DN + ES groups. In general, relatively higher intensities or frequencies of ES are likely to result in greater effects on muscle atrophy and capillary regression in early-stage DN-induced disuse. Muscle stimulation with higher-frequency ES regimens (20–100 Hz) tends to show beneficial effects of reducing the disuse-induced decreases in muscle mass, FCSA, and capillary supply [15, 25, 40–42]. However, a low-frequency ES regimen (10 Hz, 8 and 16 mA, 30 min/day, for 3 weeks) in stimulated TA muscle retarded the atrophy of denervated muscle [13]. Using the same ES conditions (10 Hz, 16 mA, 30 min/day, for one week), the present study demonstrated that daily ES treatment reduced the DN-induced decrease in FCSA. Therefore, low-frequency ES treatment might have a beneficial effect on muscle disuse atrophy.

Direct ES has been reported to increase the C/F ratio in intact fast-type dominant muscle [42, 43]. Previous studies that compared the effects of different ES frequencies on resting blood flow and capillary density reported that optimal ES frequency for angiogenesis lies in the range of 10–40 Hz [44]. Moreover, previous reports describe a significant increase in C/F ratio after only four to seven days of low-frequency ES (10 Hz) in intact TA and EDL muscles [45, 46]. Our data showed that the C/F ratios in the DN + ES groups were significantly greater than in the DN group, but not different from the control group. This suggests that daily low-frequency ES treatment effectively ameliorates muscle capillary regression in early-stage DN-induced disuse. However, our data also revealed that capillary density was unchanged following DN and ES treatment. In the present study, two-dimensional capillary density was determined by counting the total number of capillaries in a muscle section and was expressed as the number of capillaries per CSA unit. The measure of capillary density is likely subject to muscle atrophy or hypertrophy in fiber size if the C/F ratio is stable. Hudlicka et al. suggested that muscle fiber hypertrophy is always associated with reduced capillary density [47]. However, there are discrepancies among previous reports; some found that muscle fiber atrophy is accompanied by increased capillary density two and eight weeks after denervation [5], whereas others report a decrease [48]. Nevertheless, long-term denervation results in reduced capillary density and C/F ratio [49]. Some reports have also established that inactivity affects capillary density in slow and fast muscles differently [5, 50]. Thus, the incongruent results regarding capillary density might be a result of differences in the duration of disuse, degree of muscle atrophy (fiber size), and muscle type.

A potential explanation for the effects of ES on capillary proliferation involves vascular endothelial growth factor (VEGF), which is known to be an important physiological regulator of angiogenesis. Several lines of evidence suggest that VEGF may also play a vasculoprotective role [51, 52]. In animal studies, ES-induced muscle contraction or mechanical stress facilitates cytokine (myokine) production [53] and increases blood flow and capillary vascularity [54, 55]. Mechanical stress, such as shear stress and cyclic mechanical stretching, is known to induce VEGF expression [56, 57]. In fact, continuous low-voltage ES in a hind limb ischemia model of rats for five days has been reported to increase VEGF production [58]. Therefore, the enhanced blood flow and cyclic stretching generated by ES-induced muscle contractions may stimulate VEGF production and lead to angiogenesis in the ES muscles.

Furthermore, MMPs play an important role in the homeostasis and maintenance of myofiber functional integrity and regulate skeletal muscle regeneration and angiogenesis by degrading ECM components [17, 18]. Capillary sprout formation is initiated by fragmentation of the capillary basal lamina and the subsequent migration and proliferation of distinct endothelial cell populations in response to angiogenic stimuli [19, 59]. Various blood and connective tissue cells (e.g., macrophages, monocytes, and endothelial cells) secrete MMP-2 [60], and MMP-2 is involved in the degradation of type IV collagen, laminin, elastin, fibronectin, and many other ECM proteins [61]. Interestingly, an ablation study of MMP-2 reported downregulation of VEGF in the MMP-2 knockout mouse [62], and MMP-2 ablation is known to impair the growth of regenerated muscle fibers and reduce angiogenesis via decreased VEGF, Flt-1, and nNOS expression. Thus, MMP-2 may influence angiogenesis and muscle regeneration via upregulation of VEGF and its receptor Flt-1 and may also affect angiogenesis through regulation of nNOS-derived NO [21]. Our data revealed that the DN and DN + ES groups had a greater MMP-2 positive area compared to the control group, but no difference was observed between the DN and DN + ES groups. Previous studies report increased MMP-2 activity following DN and limb immobilization [24, 25] and found that MMP-2 expression is elevated to different extents depending on the intensity of chronic ES [27, 28]. On the other hand, low-intensity ES (20 Hz for 3 s followed by 6 s of rest, repeated for 20 maximal contractions, applied every 48 h for 28 days) did not alter MMP-2 activity [25]. Some discrepancies might exist among interventions with different conditions. A previous study reported that exercise-induced expression of MMP-2 is intensity dependent; high-intensity endurance exercise at approximately 70% of maximum oxygen consumption (VO_2_ max) increases MMP-2 expression, whereas low-intensity endurance exercise (approximately 50% VO_2_ max) did not alter MMP-2 expression in skeletal muscles [27]. Although we did not determine VO_2_ during daily ES treatment, our ES regimen caused approximately 25% of maximal contraction force in the TA muscle [16]. Therefore, we may not have detected de novo alteration of MMP-2 in the DN + ES rats because of the low-intensity intervention.

The present findings should be interpreted in the context of their potential limitations. First, we used young, growing rats that were undergoing skeletal remodeling and thus needed the appropriate baseline controls. Although there was no inhibition of longitudinal bone growth of the tibiae in the young DN rats over time [63], young animals usually have greater potential for musculoskeletal recovery after limb disuse [64, 65]. Our results might quantitatively differ if skeletally mature rats were used with a DN-induced disuse model. Second, our methodology precluded analysis of the activity of tissue inhibitors of metalloproteinases (TIMPs) and other pro- and antiangiogenic factors. A zymography analysis would be ideal to evaluate MMP-2 and TIMP-1 activity, and analysis of inhibitors of metalloproteinases will be the focus of future experiments. Muscle atrophy is accompanied by changes in both MMPs and TIMPs, and the ratio between these metalloproteinases may be important in determining myoblast migration and differentiation [61, 66]. Furthermore, MMPs and TIMPs play a significant role in regulating angiogenesis, which is the formation of new blood vessels [19]. For example, TIMP-2 plays an important role in ECM turnover by mediating the activity of MMPs, including MMP-2 [60]. Therefore, data on the balance between MMP-2 and TIMP-2 may provide useful information for evaluating the effects of low-frequency ES on reducing early-stage muscle atrophy and capillary regression in denervated muscles. Further studies using adult rats and other parameters and methodical techniques are needed for a more complete understanding of the relationship between ES-induced muscle contractions and capillary and muscle loss in the early stages of DN-induced muscle disuse.

In conclusion, this is the first histomorphological study to our knowledge that assesses the influence of low-frequency ES on MMP-2 immunoreactivity, fiber atrophy, and capillary regression in denervated muscle in early stages of disuse. Our results showed that FCSA and C/F ratios decreased with increased MMP-2 immunoreactivity following DN and that daily low-frequency ES treatment on denervated muscle reduced the decrease in FCSA and C/F ratios but did not alter higher levels of MMP-2 produced by DN-induced disuse. These findings suggest that direct ES has beneficial effects on early-stage DN-induced muscle disuse in the rat hind limb by ameliorating muscle atrophy and capillary regression without inducing a further increase in MMP-2 immunoreactivity. The use of direct ES to generate muscle contractions for the rehabilitation of skeletal muscles is well established, and the influence of different ES regimens on the balance of pro- and antiangiogenic factors is of great interest for future investigations as it may contribute to the understanding of the mechanisms responsible for the beneficial effects of ES.

## Figures and Tables

**Table 1:** Body weight (BW), muscle weight relative to BW, myofiber cross-sectional area (FCSA), capillary-to-fiber (C/F) ratio, capillary density (CD), and MMP-2 positive area in the TA muscles.

	CON	DN	DN + ES
BW (g)	177 ± 24	160 ± 21	160 ± 17
TA muscle weight (mg/g BW)	1.91 ± 0.18	1.23 ± 0.04^*∗∗*^	1.62 ± 0.21^††^
EDL muscle weight (mg/g BW)	0.48 ± 0.08	0.38 ± 0.03^*∗∗*^	0.38 ± 0.02^*∗*^
Sol muscle weight (mg/g BW)	0.37 ± 0.07	0.25 ± 0.04^*∗*^	0.27 ± 0.02^*∗*^
TA FCSA (*μ*m^2^)	1425 ± 272	952 ± 144^*∗*^	1365 ± 341^†^
C/F ratio	1.22 ± 0.31	0.73 ± 0.19^*∗*^	1.17 ± 0.24^†^
CD (#/mm^2^)	698 ± 272	620 ± 211	714 ± 265
MMP-2 positive area (*μ*m^2^)	275 ± 148	824 ± 341^*∗*^	1101 ± 588^*∗*^

^*∗*^ *P* < 0.05 and ^*∗∗*^*P* < 0.01 versus CON; ^†^*P* < 0.05 and ^††^*P* < 0.01 versus DN.
